# *Sapindus mukorossi* as a Bioenhancer for the Biocidal Action of Polymyxin B

**DOI:** 10.1021/acsomega.5c03035

**Published:** 2025-08-25

**Authors:** Aleksandra Makiej, Wojciech Smułek, Kamila Dubrowska, Adrian Augustyniak, Przemysław Bernat, Ewa Kaczorek

**Affiliations:** † Institute of Chemical Technology and Engineering, Faculty of Chemical Technology, 49632 Poznan University of Technology, Berdychowo 4, Poznan 60-965, Poland; ‡ Department of Chemical and Process Engineering, Faculty of Chemical Technology and Engineering, 49807 West Pomeranian University of Technology, Piastów Avenue 42, Szczecin 71-065, Poland; § Center for Advanced Materials and Manufacturing Process Engineering (CAMMPE), Piastów Avenue 42, Szczecin 71-065, Poland; ∥ Department of Industrial Microbiology and Biotechnology, Faculty of Biology and Environmental Protection, University of Lodz, Banacha 12/16, Lodz 90-237, Poland

## Abstract

Bacterial infections remain a significant health concern, necessitating the continuous exploration of novel therapeutic strategies. *Sapindus mukorossi* extract, renowned for its bioactive saponins, presents a promising approach for combating bacterial pathogens. This study investigates the potential of *S. mukorossi* extract (SM) as a bioenhancer for the polymyxin B antibiotic (PMB) against four bacterial strains: *Staphylococcus aureus* (SA), *Staphylococcus epidermidis* (SE), *Pseudomonas aeruginosa* (PA), and *Escherichia coli* (EC). Research methods were used to determine the metabolic activity of the strains and the changes in their cell membrane permeability. Furthermore, advanced microscopic techniques (confocal and transmission electron microscopy) were used to confirm the viability and visualize morphological changes within selected strains. Obtained data were also correlated with the lipidomic and fatty acid methyl ester profiles of strains subjected to the described treatments. Results indicated that the conjugated treatment of bacterial cells with PMB and SM extract demonstrated an enhancement of bacterial total membrane permeability in comparison to the treatment with PMB alone. Notably, for the *S. aureus* strain, a significant decrease in viability was noted, which can be associated with the significant (in terms of statistical analysis) increase in cell membrane permeability for cells treated with SM and PMB, compared with samples treated with PMB alone. This was further conjoined and proven by the results of the FAME and lipidomic analyses. Specifically for *S. aureus*, an increase in branched fatty acids was detected in cells exposed to SM and SM + PMB. Additionally, the lipidomic analysis revealed notable membrane remodeling, characterized by an increase in lysyl-phosphatidylglycerol and diglucosyldiglyceride and a decrease in phosphatidylglycerol, in samples treated with SM and SM + PMB compared to the control group. This study underscores the potential of *S. mukorossi* extract as an enhancing agent to PMB in combination therapies against bacterial infections, paving the way for further investigations into its mechanistic insights and clinical applications.

## Graphical abstract

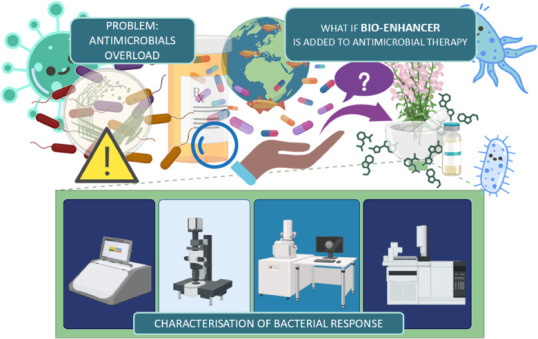

## 1 Introduction

Antimicrobial resistance (AMR) has emerged as one of the most pressing environmental and public health challenges of the 21st century. The widespread use of antibiotics in human medicine, agriculture, and aquaculture has significantly contributed to the environmental dissemination of antibiotic-resistant bacteria (ARB) and antibiotic-resistant genes (ARGs). These contaminants, often referred to as emerging pollutants, are transported into soil, water, and other ecosystems, where they pose risks to human, animal, and plant health.^1–3^

In this context, natural compounds capable of mitigating antibiotic use or enhancing their efficacy represent an environmentally sustainable approach to combating AMR. In the quest to boost the efficacy of already existing antibiotics, the exploration of augmenting interactions between natural compounds and antimicrobial agents has arisen as a compelling direction of research. Among these, *S. mukorossi*, commonly known as the soapberry or reetha, has garnered attention for its multifaceted bioactivities, ranging from its traditional use in cleansing agents to its potential in modern pharmacotherapy.^4^ *S. mukorossi*, a member of the *Sapindaceae* family, is native to the subtropical regions of Asia and is renowned for its rich reservoir of bioactive compounds.^5^ Traditionally recognized for its detergent properties, recent scientific ventures have unveiled its diverse pharmacological activities, including antimicrobial, anti-inflammatory, and immunomodulatory effects.^6^ Notably, the fruit from *S. mukorossi* holds significant medicinal value, being employed in the treatment of diverse ailments such as epilepsy, pimples, migraines, eczema, and psoriasis, alongside exhibiting insecticidal properties for the removal of scalp lice.^7,8^ Moreover, the powdered seeds find application in remedying dental caries, arthritis, the common cold, constipation, and nausea, while the leaves are employed in baths for alleviating joint pain, and the roots are utilized in the treatment of gout and rheumatism.^9^

Interestingly, saponins from various plant sources, including *S. mukorossi*, have also been explored as biosurfactants for environmental remediation, particularly in washing out heavy metals from polluted environments.^10–12^ Within this spectrum, its role as a bioenhancer for the potent antimicrobial agent stands out as a beacon of innovation.^13^ At the forefront of this paradigm shift is the antibacterial action enhancement of *S. mukorossi* extracts in combination with polymyxin B, a last-resort antibiotic known for its efficacy against Gram-negative bacteria. While polymyxin B exerts its antimicrobial action primarily through disruption of the bacterial cell membrane, we assumed that SM compounds might be shown to augment this effect, thereby enhancing the susceptibility of bacteria to PMB. However, in our previous studies,^13^ we have not explored this effect from a mechanistic perspective. The current research therefore elucidates the novel field of utilizing *S. mukorossi* extracts to magnify the biocidal efficacy of PMB, offering insights into its *in vitro* mechanisms of action on *S. aureus*, *S. epidermidis*, *P. aeruginosa*, and *E. coli*. In this work, findings not only demonstrate the potentiating antimicrobial action of SM extract and polymyxin B against pathogenic bacterial strains but also highlight the potential for reducing the environmental footprint of PMB. Thereupon, our outcomes underscore the role of *S. mukorossi* as a sustainable bioenhancer, paving the way for biocidal applications that align with environmental safety and public health priorities.

## 2 Materials and Methods

### 2.1 Chemicals

The high-purity polypeptide antibiotic polymyxin B was sourced from Alchem Grupa Sp. z o.o. (Toruń, Poland). *Sapindus mukorossi* extract was prepared as described previously by our group.^13^ Additional chemicals, including MTT reagent (3-(4,5-dimethylthiazol-2-yl)-2,5-diphenyltetrazolium bromide), Crystal violet, and the LIVE/DEAD BacLight Bacterial Viability Kit, were obtained from Thermo Fisher Scientific (Waltham, MA, USA). Nutrient agar, nutrient broth, and other microbiological supplements were supplied by BTL Sp. z o.o. (Łódź, Poland). The *Pseudomonas aeruginosa* (PCM 2720) and *Escherichia coli* (PCM 2857) strains were acquired from the Polish Collection of Microorganisms (Wrocław, Poland), while *Staphylococcus aureus* (ATCC 25923) and *Staphylococcus epidermidis* (ATCC 12228) were obtained from the ATCC collections (Manassas, United States).

### 2.2 Methods

#### 2.2.1 Metabolic Activity and Cell’s Membrane Properties

The metabolic activities of *P. aeruginosa*, *E. coli*, *S. aureus*, and *S. epidermidis* were determined using the colorimetric MTT assay, as per the protocol of Wang et al.^14^ The total cell membrane permeability of each strain was assessed using the crystal violet assay, following the method described by Devi et al.^15^. Additionally, the cell surface hydrophobicity of the strains was quantified using the Congo red assay according to Ambalam et al.^16^ Each of the bacterial strains was initially grown on nutrient agar plates and then cultured in nutrient broth, incubating for 24 h at 120 rpm to reach the exponential growth phase. The cultures were centrifuged at 4500 rcf, resuspended in a PMB solution at a neutral pH, and adjusted to a final bacterial concentration of 1 × 10^8^ cfu/mL. All experiments with microorganisms were conducted after exposing the bacterial cells to polymyxin B (PMB, 100 mg/mL), *S. mukorossi* (SM, 5 mg/L), or their combination for 24 h. Concentrations of 100 mg/mL PMB and 5 mg/mL SM were chosen based on our previous optimization study.^13^ Each assay was performed on three independent biological replicates, and each biological replicate was measured in triplicate (technical replicates).

#### 2.2.2 Bacterial Surface Morphology Observations by the Confocal Microscope, AFM, and TEM

Surface morphology was characterized using an atomic force microscope (Park NX10, Park Systems, Suwon, South Korea) as per Pacholak et al.^17^ Bacterial morphology was further observed using a Leica Stellaris 5 WLL confocal microscope (Wetzlar, Germany) with the LIVE/DEAD BacLight Bacterial Viability Kit, following the protocol by Honselmann genannt Humme et al.^18^ with minor modifications including sample observations of cells immobilized on dark filters (0.22 μm Polycarbonate Membrane Filter, Merck, Germany). Observations under TEM, performed with a Jeol 1200 EX II instrument (Jeol, USA), were done according to Pacholak et al.^19^ and allowed for the observation of ultrastructural details, complementing AFM and fluorescence microscopy findings.

#### 2.2.3 Bacterial Lipids Analyses

The procedure was conducted as in Wójcik-Bojek et al.^20^ and Jasińska et al.^21^ with modifications including mechanical bead milling for lipid extraction, integrated fatty acid methyl esters (FAMEs), and lipidomic profiling to assess membrane composition and dynamics.

### 2.3 Statistical Analysis

The data presented represent the averaged results from at least three independent experimental replicates, expressed as mean values. Statistical analysis was conducted using a one-way analysis of variance (ANOVA), followed by Tukey’s post hoc test (α = 0.05) to determine the significance of differences between means, with a significance level set at *p* < 0.05. Microsoft Office Excel 2019 was used for preliminary statistical calculations, while more detailed analyses were performed using GraphPad Prism (version 8.4.3, GraphPad Software, La Jolla, California, USA, www.graphpad.com). Further statistical tests, including Student’s *t*-tests, were executed with thresholds for significance defined as follows: *p* < 0.05 (*), *p* < 0.01 (**), and *p* < 0.001 (***). Tables S1 and S2 provide the adjusted *p*-values from all statistical analyses.

## 3 Results

### 3.1 Bacterial Metabolic Activity and Membrane Permeability Assessment

The treatment with the antibiotic polymyxin B (PMB) resulted in the greatest inhibition of growth across all tested bacterial strains, as demonstrated in Figure 1. The antibiotic exhibited the most significant impact on the metabolic activity of both *P. aeruginosa* (a Gram-negative bacterium) and *S. aureus* (a Gram-positive bacterium). Similarly, the extract from *S. mukorossi* and its combination with the antibiotic showed notable inhibitory effects on the growth of these strains. Although the plant extract did not surpass the antibiotic in effectiveness, it still achieved 64% and 69% reductions in metabolic activity for *P. aeruginosa* and *S. aureus*, respectively. Treatment with the combined polymyxin B and plant extract resulted in a level of metabolic activity inhibition that was less potent than the antibiotic alone but more pronounced than the plant extract alone.

**Figure 1:**
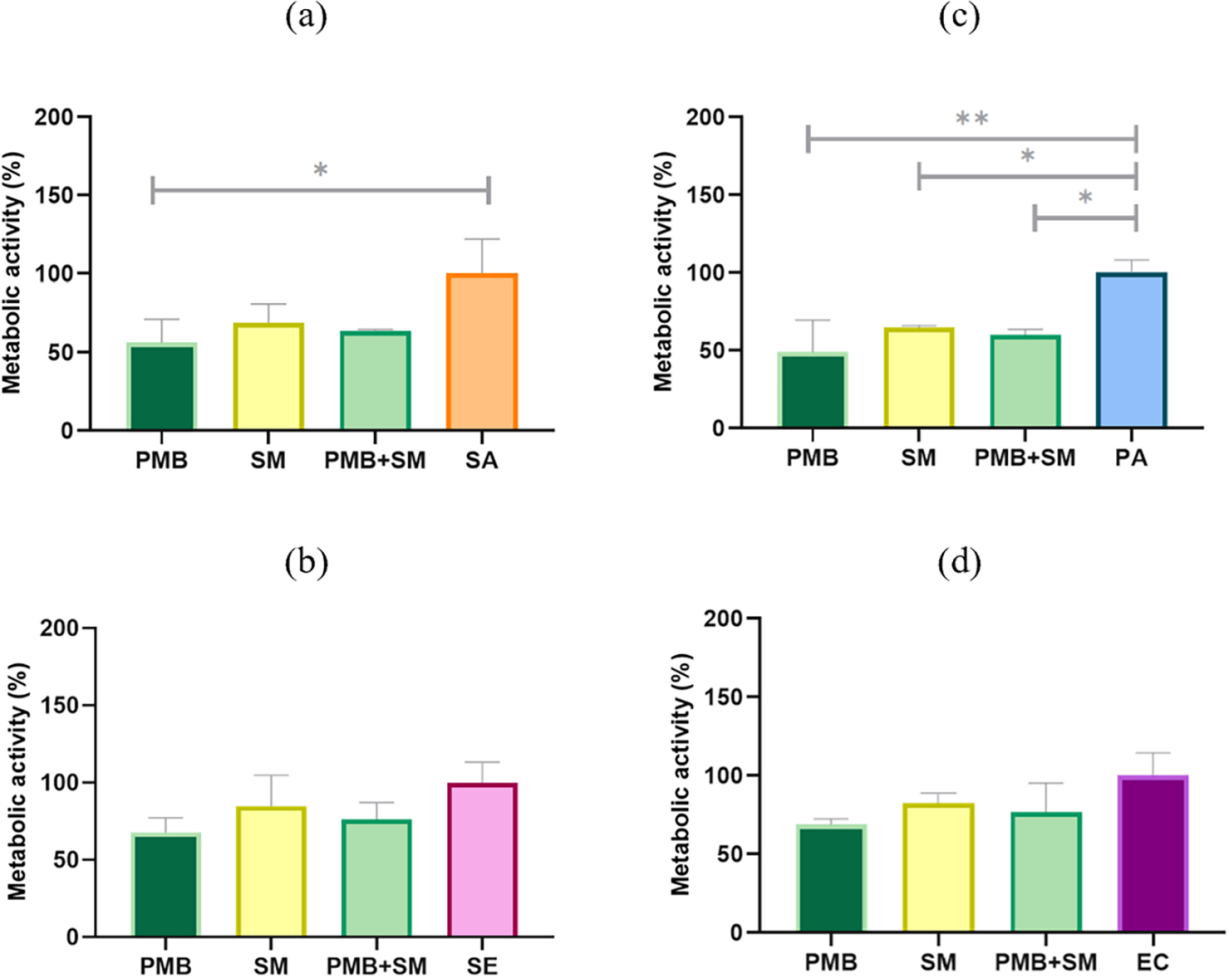
Metabolic activity of selected microbial strains under stress conditions (100% represents the control, untreated samples); Gram-positive strains: (a) SA – *S. aureus*, (b) SE – *S. epidermidis*, and Gram-negative strains: (c) PA – *P. aeruginosa*, (d) EC – *E. coli*; PMB – polymyxin B (100 mg/mL), SM – *S. mukorossi* (5 mg/mL). Detailed information from the statistical analysis can be found in Table S1 a–d.

Moreover, the assessment of total bacterial membrane permeability revealed that polymyxin B, when used alone, did not significantly alter the permeability in any of the tested strains (Figure 2). In contrast, the *S. mukorossi* extract independently increased membrane permeability by a few percentage points across all strains, with a slightly less pronounced effect observed when combined with polymyxin B.

**Figure 2:**
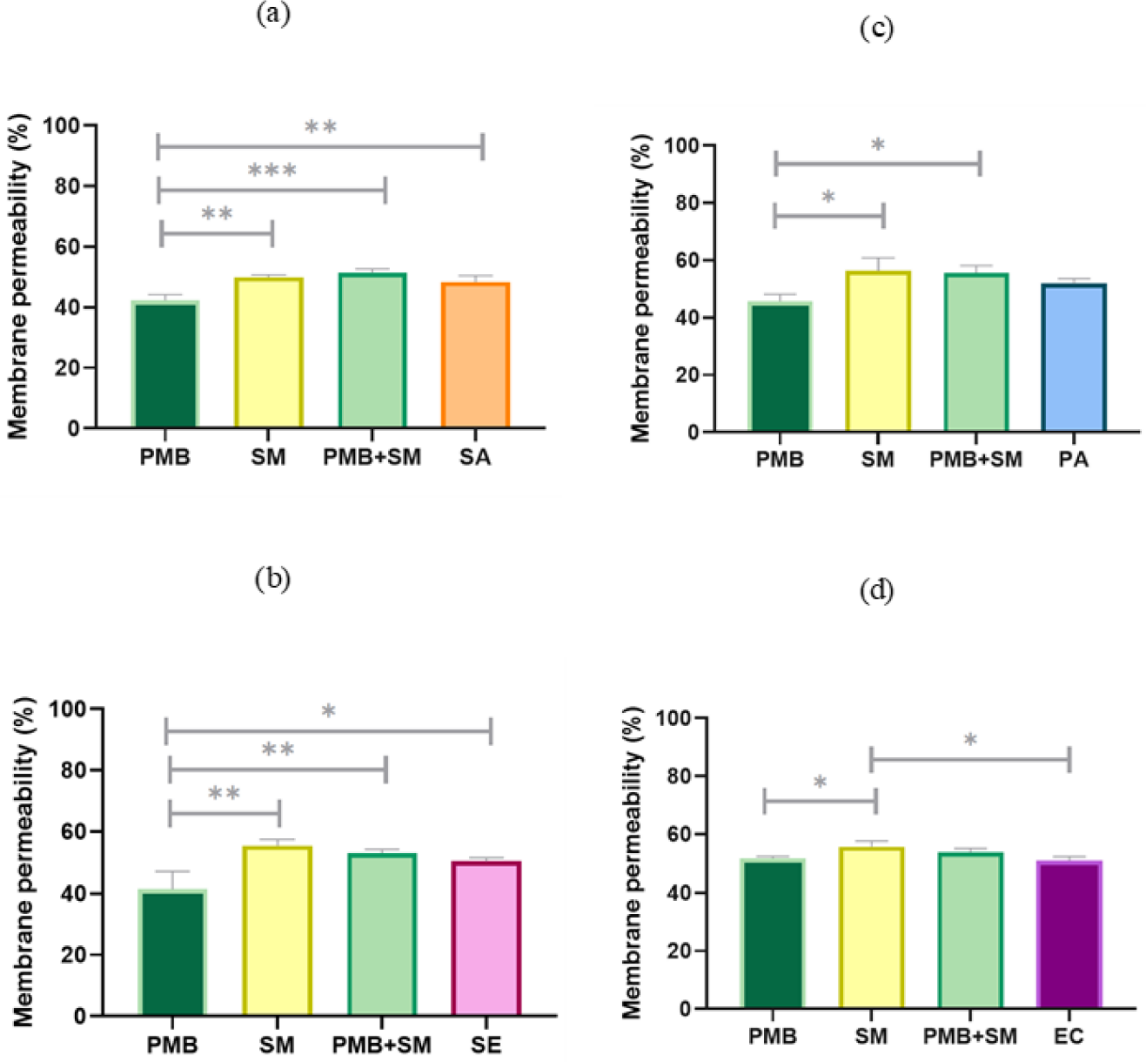
Total membrane permeability of selected microbial strains under stress conditions in comparison to bacterial untreated control samples; Gram-positive strains: (a) SA – *S. aureus*, (b) SE – *S. epidermidis*, and Gram-negative strains: (c) PA – *P. aeruginosa*, (d) EC – *E. coli*; PMB – polymyxin B (100 mg/mL), SM – *S. mukorossi* (5 mg/mL). Detailed information from the statistical analysis can be found in Table S2 a–d.

### 3.2 AFM Analysis

As seen from the images in Figure 3, in the untreated control groups, cells generally display intact and smooth surfaces, indicating a typical healthy state. Upon exposure to PMB, notable surface disruptions and irregularities are observed, particularly in PA and EC, which exhibit membrane blebbing and cell surface roughness. This suggests PMB’s potent membrane-disruptive action. SM treatment also induces noticeable changes, albeit more subtle than PMB, such as slight surface roughening and the appearance of minor pits, indicating partial membrane destabilization.

**Figure 3:**
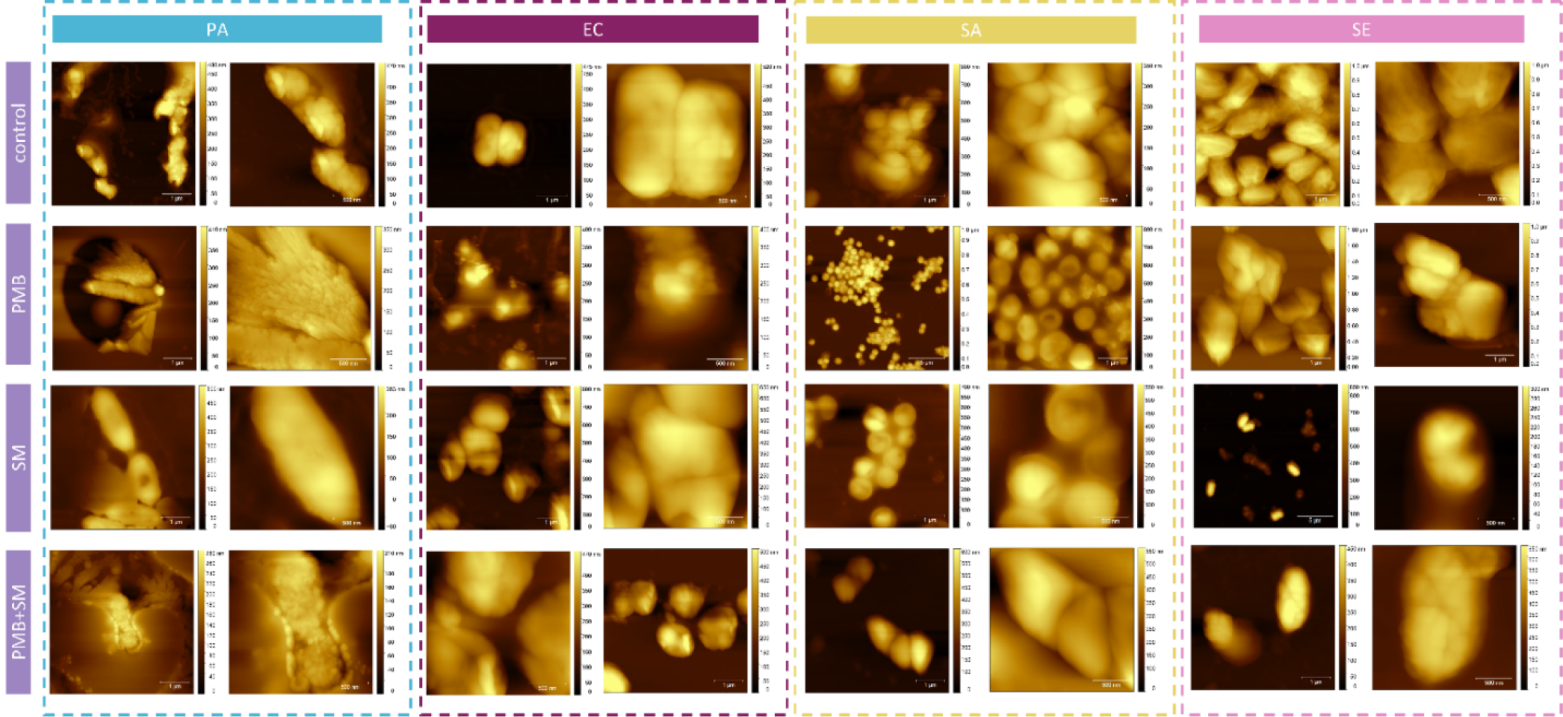
AFM images of selected microbial strains after treatment in comparison to bacterial untreated control samples; Gram-positive strains: SA – *S. aureus*, SE – *S. epidermidis*, and Gram-negative strains: PA – *P. aeruginosa*, EC – *E. coli*; treatments: PMB – polymyxin B (100 mg/mL), SM – *S. mukorossi* (5 mg/mL), independently and in conjunction.

The most pronounced morphological alterations are seen in the combined PMB + SM treatment across all bacterial types. These cells often display significant structural damage, including membrane collapse, pronounced surface roughness, and, in some cases, complete disintegration of cell shape. This suggests an augmented effect of the combined treatment, leading to severe disruption of bacterial cell membranes and a potential leakage of intracellular contents.

What is more, the calculations from AFM data analysis (Table 1) revealed distinct morphological alterations in bacterial cells following treatment with polymyxin B (PMB), *S. mukorossi* (SM), and their combination (PMB + SM). Across *E. coli* and *P. aeruginosa*, treatments generally resulted in decreased cell dimensions (length, width, and height) compared to the untreated control, with notable shrinkage in cell length observed under the influence of PMB + SM. In *P. aeruginosa*, the treatment also led to an increase in surface roughness, particularly in the PMB + SM group, indicating significant cell surface disruption. For *S. aureus*, treatment with PMB and SM led to a reduction in cell length and width, along with a decrease in surface roughness, suggesting potential cell wall compromise. Conversely, *S. epidermidis* showed increased cell length under PMB + SM treatment, with a concurrent reduction in height and surface roughness, indicating differential stress responses among the bacterial species.

**Table 1:** AFM Data Analysis of Microbial Structure after Treatment in Comparison to Bacterial Untreated Control Samples Gram-positive bacteria tested: SA – *S. aureus*, SE – *S. epidermidis*, and Gram-negative bacteria tested: PA – *P. aeruginosa*, EC – *E. coli*; treatments: PMB – polymyxin B (100 mg/mL), SM – *S. mukorossi* (5 mg/mL), independently and in conjunction; l – length; w – width; h – height; Ra – arithmetic average roughness; Rq – root mean square roughness; R3z – average of three maximum peak-to-valley heights; Ry = Rmax – maximum height of the profile; Wq – root mean square width.

	*E. coli*				*P. aeruginosa*			
	ctrl	PMB	SM	PMB + SM	ctrl	PMB	SM	PMB + SM
**l**	1591	1277	1493	1265	965	2172	1777	1780
**w**	900	821	972	908	524	786	912	791
**h**	432	302	556	387	482	251	261	397
**Ra**	4	3	5	6	8	2	2	3
**Rq**	5	3	6	7	9	3	2	3
**R3z**	14	10	18	17	25	8	6	9
**Ry = Rmax**	19	12	23	18	28	14	8	13
**Wq**	33	29	54	49	41	16	23	26

	*S. aureus*				*S. epidermidis*			
	ctrl	PMB	SM	PMB + SM	ctrl	PMB	SM	PMB + SM
**l**	860	863	802	700	1617	1465	889	2286
**w**	841	874	725	629	886	866	490	998
**h**	438	600	455	482	736	754	162	229
**Ra**	6	4	5	2	7	5	3	2
**Rq**	8	4	5	2	8	5	4	2
**R3z**	21	9	15	5	24	17	11	5
**Ry = Rmax**	17	9	11	5	29	16	11	9
**Wq**	34	31	24	7	48	48	16	8

Furthermore, the AFM analysis of the bacterial samples revealed significant changes in mechanical properties upon all tested treatments (Table 2). For *E. coli* and *P. aeruginosa*, treatments generally led to an increase in cell adhesion energy (Ea) and elasticity modulus (Me), particularly in the PMB + SM groups, indicating increased cell rigidity and altered surface characteristics. *E. coli* showed reduced cell adhesion force (Fa) under all treatments, suggesting smoother or less interactive surfaces, while *P. aeruginosa* exhibited a marked increase in Fa under PMB + SM, indicating heightened surface interactions. For *S. aureus*, the PMB + SM treatment notably increased both the levels of Ea and Me, suggesting enhanced cell rigidity and structural alterations. In contrast, *S. epidermidis* exhibited decreased Ea and Fa across all treatments, indicating smoother surfaces or reduced adhesion properties, with a notable reduction in Me under SM, PMB, and PMB + SM, implying increased membrane flexibility.

**Table 2:** Mechanical Data Analysis from AFM Measurements for Selected Microbial Strains after Treatment in Comparison to Bacterial Untreated Control Samples Gram-positive strains: SA – *S. aureus*, SE – *S. epidermidis*, and Gram-negative strains: PA – *P. aeruginosa*, EC – *E. coli*; treatments: PMB – polymyxin B (100 mg/mL), SM – *S. mukorossi* (5 mg/mL), independently and in conjunction; Ea (aJ) – adhesion energy per unit area; Fa (nN) – adhesion force; Me (GPa) – elastic modulus.

	*E. coli*				*P. aeruginosa*			
	ctrl	PMB	SM	PMB + SM	ctrl	PMB	SM	PMB + SM
**Ea(aJ)**	579.60	683.93	207.22	692.93	54.63	57.41	40.37	593.97
**Fa(nN)**	74.05	44.24	22.83	47.26	13.19	17.15	8.85	58.67
**Me(Gpa)**	0.91	0.37	1.01	0.59	0.90	1.02	1.20	2.13

	*S. aureus*				*S. epidermidis*			
	ctrl	PMB	SM	PMB + SM	ctrl	PMB	SM	PMB + SM
**Ea(aJ)**	271.83	108.95	172.53	441.92	146.14	91.19	68.69	57.91
**Fa(nN)**	30.72	17.92	41.28	36.42	30.75	17.15	15.84	12.60
**Me(Gpa)**	0.75	0.80	1.41	1.06	2.19	1.84	1.00	1.16

Overall, the AFM analysis results indicate that both Gram-negative and Gram-positive bacteria exhibit varied stress responses in membrane integrity and mechanical properties when exposed to PMB, SM, and their combination. The observed changes in morphology and mechanical properties likely reflect adaptations to maintain cellular integrity under stress, with variations dependent on the specific bacterial strain and the nature of the treatment. These AFM-derived metrics serve as quantitative proxies for membrane disruption and complement our qualitative confocal microscopy observations.

### 3.3 Confocal Laser Scanning Microscopy

Images from CLSM showed significantly reduced viability of *P. aeruginosa* (PA) cells under the influence of antibiotic treatment (Figure 4). Treatment with *Sapindus mukorossi* extract alone did not considerably diminish cells’ viability, and their survival was possible. On the other hand, treatment with the conjugated system (polymyxin B together with plant extract) gave an image of *P. aeruginosa* cells with a strong signal in the red channel, which showed that the cells either had their membranes disturbed or were not viable. In the case of *E. coli* (EC), marked viability was observed after treating the cells with *S. mukorossi* extract alone (interestingly, the cells in this case gave an even greater viability response in the green channel than in the case of the control alone). As for *E. coli* samples that were treated with conjugated antibiotic-plant extract systems, the cells did not show any viability response, and only dead cells were visualized. *S. aureus* cells showed a strong lethality response to the antibiotic alone. A slight viability of SA cells was also observed under the influence of the *S. mukorossi* extract alone, while the viability was significantly reduced after treatment with the conjugated systems. *S. epidermidis* (SE) cells showed a clear signal in the red channel indicating decreased viability under the influence of the antibiotic, but also a clear green signal under the influence of the plant-derived extract itself, which indicated survivability of cells in this case. Treatment of SE cells with conjugated systems (antibiotic-plant extract) resulted in imaging both live and dead cells.

**Figure 4:**
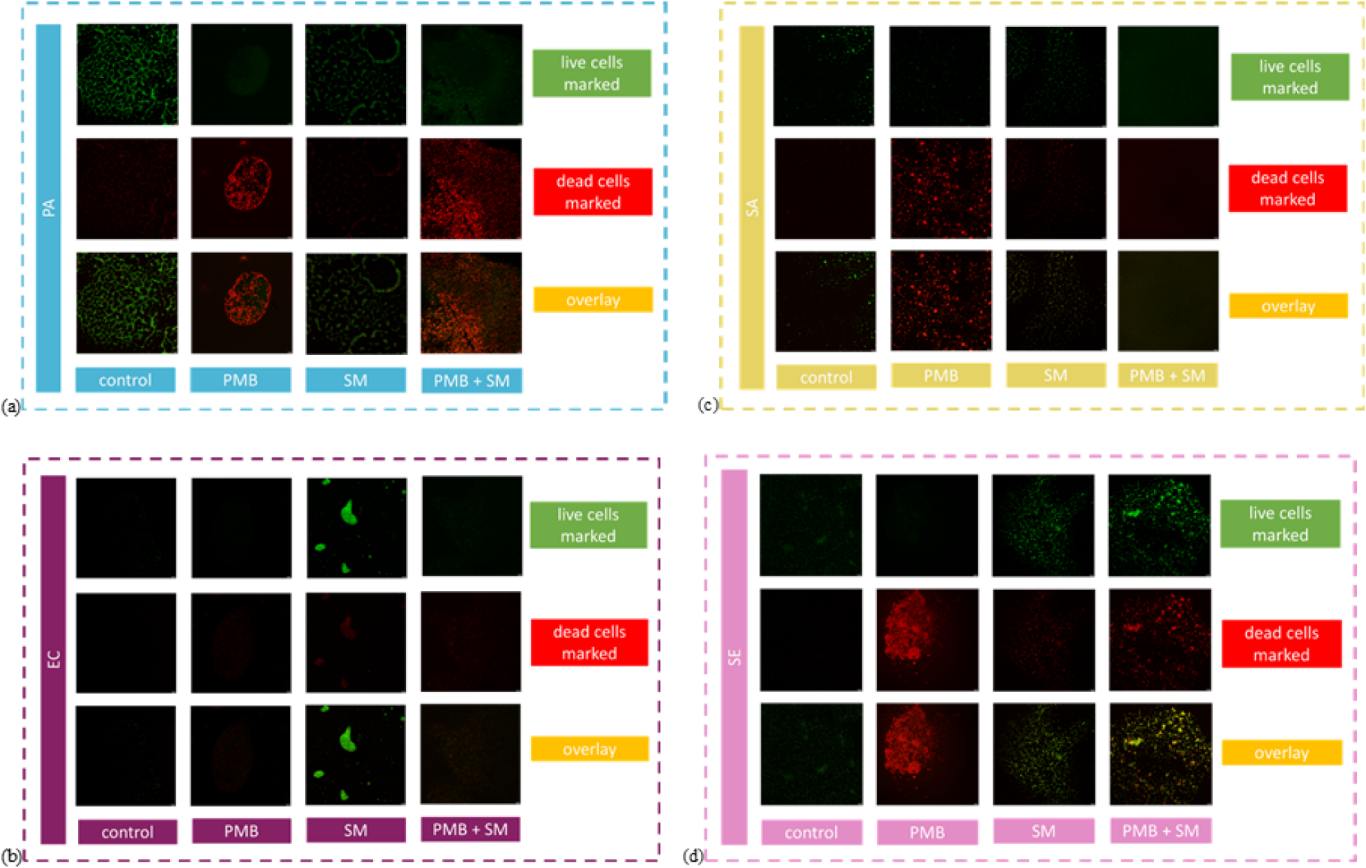
Images with marked live and dead bacterial strains after treatment in comparison to control samples (bacteria untreated); Gram-negative strains: (a) PA – *P. aeruginosa*, (b) EC – *E. coli*, and Gram-positive strains: (c) SA – *S. aureus*, (d) SE – *S. epidermidis*; treatments: PMB – polymyxin B (100 mg/mL), SM – *S. mukorossi* (5 mg/mL). Treatments were provided individually and in conjunction. Images were obtained from confocal laser scanning microscope.

### 3.4 Transmission Electron Microscope

TEM analysis was performed on selected most promising representatives of bacterial cells (Figure 5), that is, the Gram-positive bacterium *S. aureus* (SA) and the Gram-negative bacterium *P. aeruginosa* (PA). The antibiotic itself, in the case of SA, caused thinning of the outer bacterial wall, while in the case of PA, it caused visible disruption of the outer membrane. In the case of treating SA cells with the *S. mukorossi* extract alone, it resulted in the formation of gaps in the SA cell wall, while in the case of PA cells, the structure of the outer membrane was relatively smooth, although sometimes ruptures in its membrane were also noted. The greatest noticeable disruption of the structure of the outer membrane in both tested strains was observed in the case of sample (SA and PA) conjugated systems (antibiotic-plant extract).

**Figure 5:**
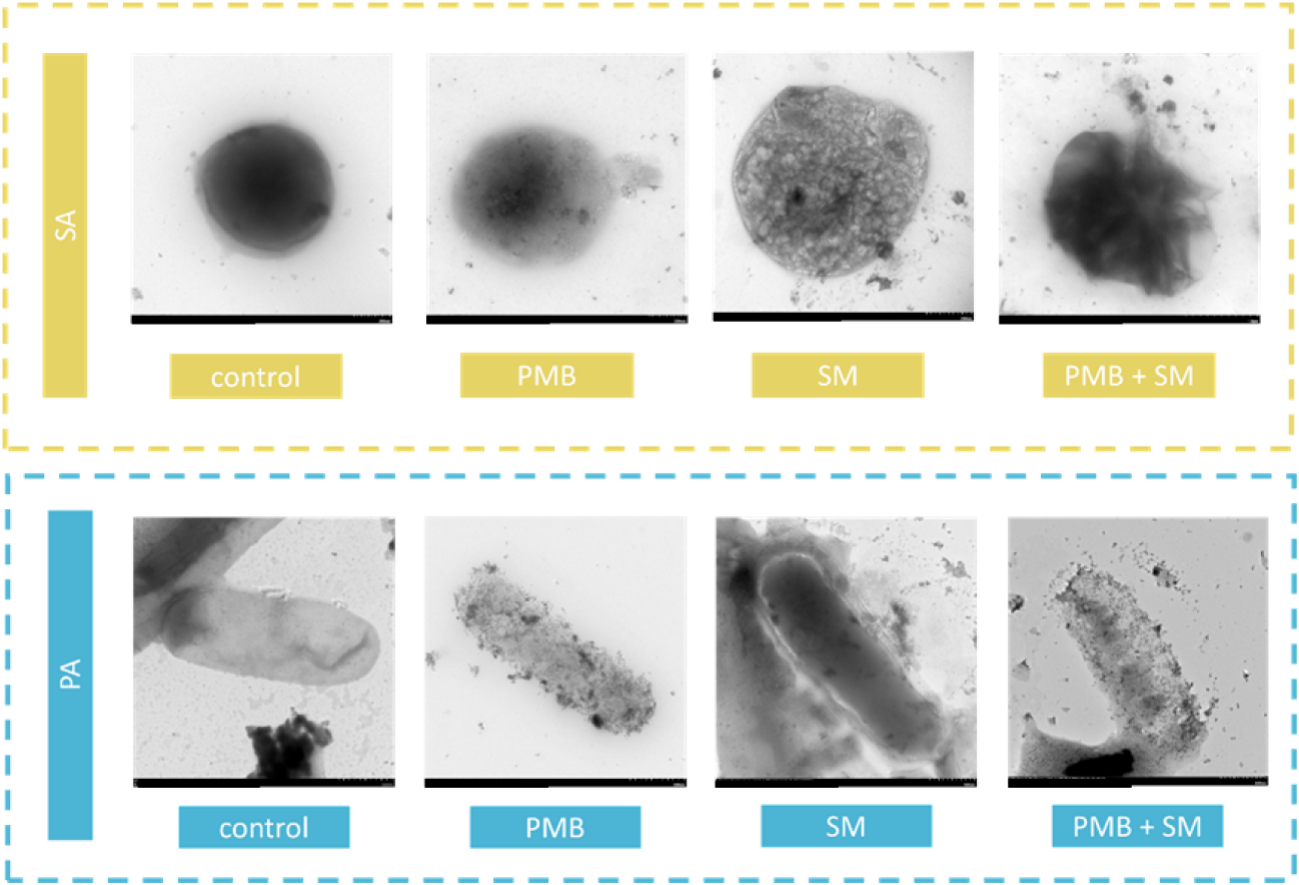
Images of bacterial cells after treatment in comparison to control samples (untreated bacteria); SA – *S. aureus*, PA – *P. aeruginosa*; treatments: PMB – polymyxin B (100 mg/mL), SM – *S. mukorossi* (5 mg/mL). Treatments were supplied individually and in conjunction. Images were obtained from a transmission electron microscope.

### 3.5 Fatty Acid Methyl Esters and Lipidomic Analysis

Results from FAME analysis (Figure 6) show that control samples (untreated bacteria) exhibit a relatively balanced composition of saturated, unsaturated, and branched fatty acids. Treatment with PMB and SM, either alone or in combination, generally led to an increased proportion of saturated fatty acids (e.g., 14:0, 16:0, 18:0), indicating a trend toward reduced membrane fluidity and improved stability under stress conditions. Notably, for *S. aureus* (SA), the quantity of branched-chain fatty acids (i.e., anteisoC15:0, anteisoC17:0) increased after the treatment with PMB, as well as with SM, and SM together with PMB, compared to the untreated control *S. aureus* strain. Conversely, the level of unsaturated fatty acids (e.g., 18:1) in Gram-negative strains decreased, particularly under the combined treatment, correlating with a potential decrease in membrane fluidity and an increase in membrane rigidity. These shifts in fatty acid composition highlight the bacteria’s adaptive responses to the stress imposed by the antimicrobial agents.

**Figure 6:**
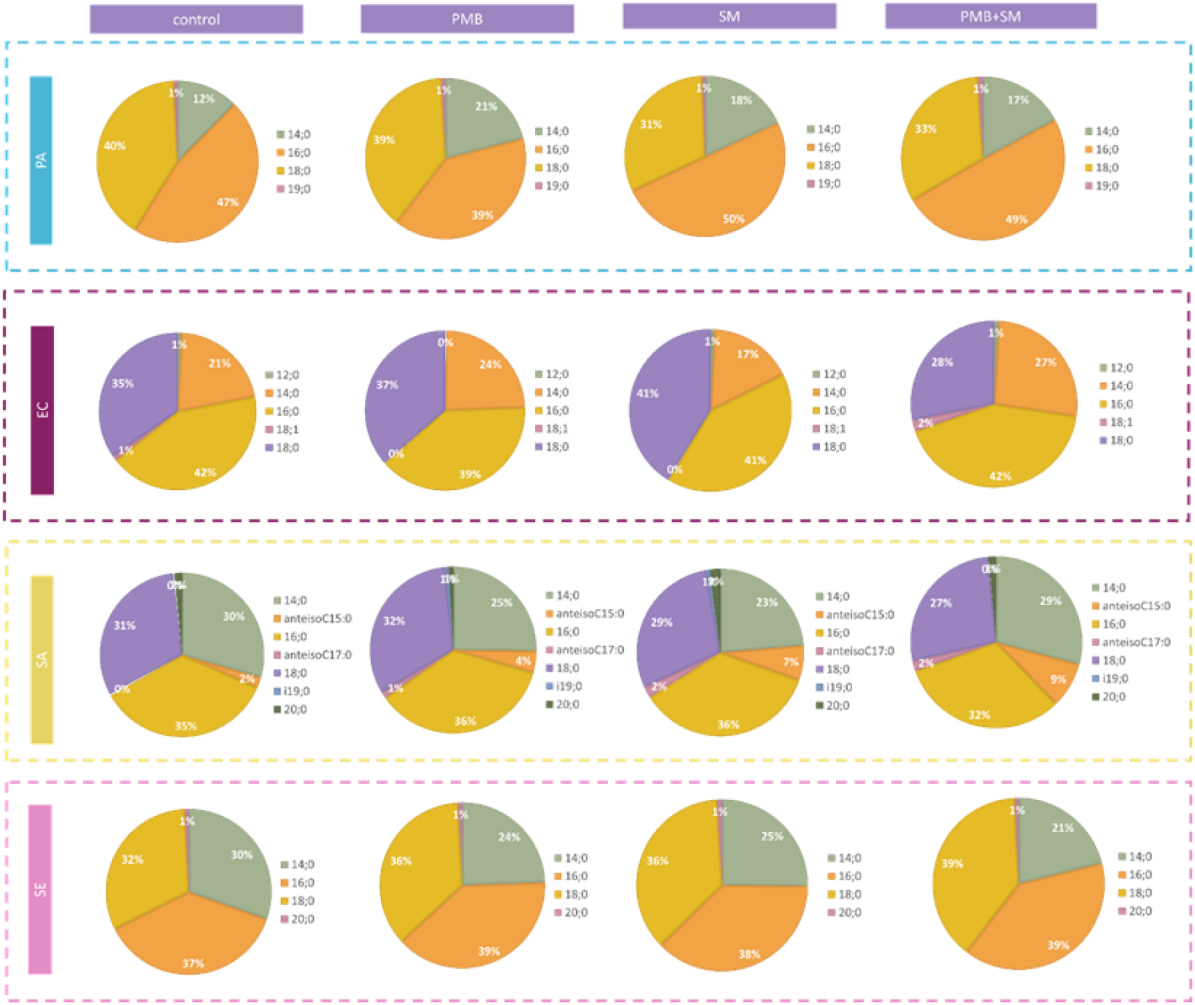
FAME analysis of bacterial membranes after treatment in comparison to control samples (untreated bacteria); Gram-positive strains: SA – *S. aureus*, SE – *S. epidermidis*, and Gram-negative strains: PA – *P. aeruginosa*, EC – *E. coli*; treatments: PMB – polymyxin B (100 mg/mL), SM – *S. mukorossi* (5 mg/mL); treatments provided individually and in conjunction. FAME abbreviations: 12:0 – Lauric acid (Dodecanoic acid); 14:0 – Myristic acid (Tetradecanoic acid); anteisoC15:0 – Anteiso-Pentadecanoic acid (12-Methyltetradecanoic acid); 16:0 – Palmitic acid (Hexadecanoic acid); anteisoC17:0 – Anteiso-Heptadecanoic acid (14-Methylheptadecanoic acid); 18:0 – Stearic acid (Octadecanoic acid); 18:1 – Oleic acid (Octadecenoic acid); i19:0 – Iso-Nonadecanoic acid (17-Methyloctadecanoic acid); 19:0 – Nonadecanoic acid (Nonadecylic acid); 20:0 – Arachidic acid (Eicosanoic acid).

In further lipidomic analysis (Figures 7 and S1), in the Gram-negative strains, an increase in phosphatidylethanolamine (PE) levels was observed across almost all treatments, particularly pronounced in PMB-treated samples. This increase suggests enhanced membrane fluidity, potentially as an adaptive response to maintain membrane integrity under stress. The rise in phosphatidylglycerol (PG) levels, especially in PA treated with PMB and SM, indicates a mechanism to stabilize membrane proteins and support membrane potential, which is crucial for maintaining bacterial viability under stress.

**Figure 7:**
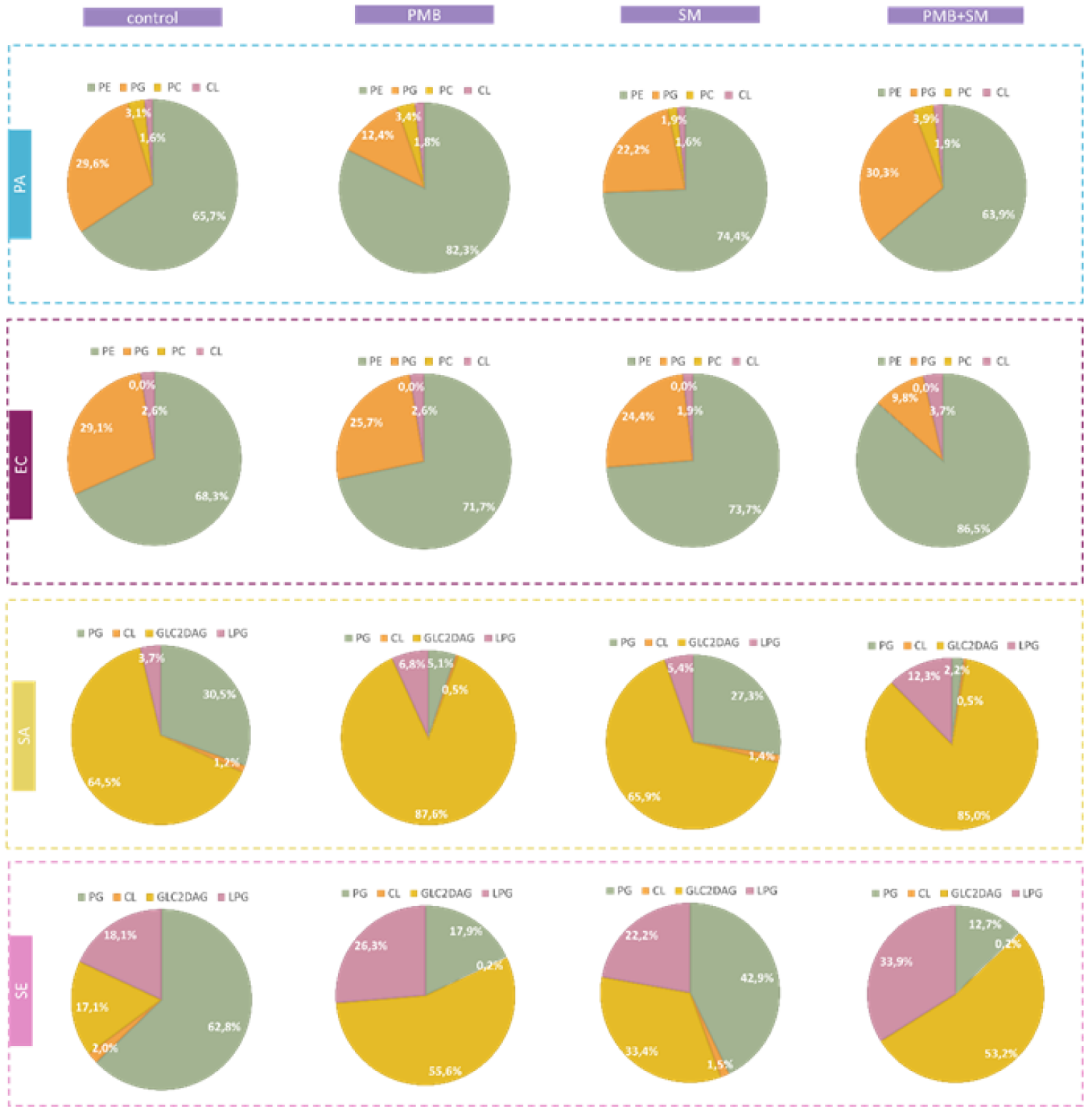
Lipidomic analysis of bacterial membranes after treatment in comparison to control samples (untreated bacteria); Gram-positive strains: SA – *S. aureus*, SE – *S. epidermidis*, and Gram-negative strains: PA – *P. aeruginosa*, EC – *E. coli*; treatments: PMB – polymyxin B (100 mg/mL), SM – *S. mukorossi* (5 mg/mL); treatments provided individually and in conjunction. Lipid abbreviations: PE: Phosphatidylethanolamine; PG: Phosphatidylglycerol; PC: Phosphatidylcholine; CL: Cardiolipin; GL2DAG: Diglucosyldiglyceride; LPG: Lysyl-phosphatidyl-glycerol. Detailed data have been included in Figure S1.

In contrast, a significant increase in cardiolipin (CL) levels was observed in SA and SE treated with PMB and SM, indicating an adaptation related to membrane stability and respiratory function, which is critical under stress conditions. The increased levels of diglucosyldiglyceride (GL2DAG) in these Gram-positive bacteria suggest their role in maintaining the functionality of the membrane under stress conditions. The observed decrease in CL and PG in SE under combined treatment could reflect a disruption in membrane biosynthesis pathways, potentially compromising the cell viability and membrane stability. As observed in our studies for SA and SE strains, an increase in membrane permeability leading to a decrease in those cells’ metabolic activity was observed when those cells were treated with SM and SM + PMB compared to those samples treated with PMB alone.

Additionally, the elevated levels of lysylphosphatidylglycerol (LPG), esterified PG with lysine, may serve to modulate the cytoplasmic membrane change in SE under stress treatments, hinting at a complex lipid remodeling response. Overall, our findings highlight bacterial adaptation strategies at the lipidomic level, where alterations in specific lipid classes reflect attempts to maintain membrane integrity, fluidity, and functionality under adverse conditions induced by antimicrobial agents.

## 4 Discussion

Based on the results obtained from our study, it is evident that both polymyxin B (PMB) antibiotic and *S. mukorossi* (SM) extract individually exerted inhibitory effects on the growth of bacterial strains, albeit with varying degrees of efficacy. The greatest growth inhibition across all tested bacterial strains was observed with PMB alone, indicating its potent antimicrobial activity. Notably, PMB demonstrated a significant impact on the metabolic activity of *P. aeruginosa* (PA) and *S. aureus* (SA), both Gram-negative and Gram-positive bacteria, respectively, aligning with previous findings.^22–24^ Conversely, the plant extract alone exhibited moderate inhibition of bacterial growth, with notable effects observed against PA and SA. These results are consistent with prior reports highlighting the antimicrobial potential of *S. mukorossi* extract against various bacterial strains.^25–28^

Interestingly, the combination of PMB with *S. mukorossi* extract yielded augmented effects, which is particularly evident in enhancing the total membrane permeability of bacterial strains. While PMB alone did not significantly affect membrane permeability, the extract alone and in conjunction with PMB demonstrated an increase in membrane permeability across all tested strains. This suggests a complementary action of the plant extract in potentiating the membrane-disrupting effects of PMB, corroborating findings from similar studies on bacterial pathogens.^29,30^

Microscopic analysis further elucidated the differential responses of bacterial cells to the treatments. Confocal imaging revealed distinct changes in the viability of cells, with PMB treatment leading to an increased death rate in PA, while the plant extract alone exhibited less pronounced effects on cell viability, consistent with observations from previous studies.^31,32^ Notably, the conjugated treatment induced a phase of death in PA cells, suggesting an augmented action in enhancing bactericidal effects.

TEM analysis provided insights into the structural alterations induced by the treatments. PMB treatment resulted in thinning of the bacterial cell wall in SA and disruption of the outer membrane in PA, consistent with its mechanism of action. Interestingly, treatment with the plant extract alone led to the formation of gaps in the cell wall, indicative of membrane perturbation. The most noticeable disruption of the membrane structure was observed in cells treated with the conjugated PMB-plant extract system, highlighting the augmented effects on membrane integrity.

An additional perspective is provided by lipidometric analysis. Comparing the fatty acid profile, there were no significant differences between cells from the reference sample and cells treated with the antibiotic and/or saponins. Only for SA and EC could it be observed that the copresence of *S. mukorossi* extract and PMB resulted in an increased proportion of fatty acids with shorter chains. Phospholipid analysis, on the other hand, shows that for PA and SA, more significant changes are induced by the presence of the antibiotic, while for EC only the presence of a plant surfactant and PMB led to the key change, which was the reduction of phosphatidylglycerol in favor of phosphatidylethanolamine.

There are few reports in the literature on the effects of saponins on the phospholipid profile, although they show that saponins interact with phospholipids,^33,34^ but in the complex structure of the bacterial membrane, they are unlikely to fully intercalate, and their lipid parts penetrate the membrane structure.^35^ The marked increase in LPG levels in SM + PMB-treated *S. aureus* (Figure 7) parallels the enhanced total membrane permeability measured in Figure 2a, supporting a causal link between lipid remodeling and membrane disruption. Likewise, the decrease in PG species in *E. coli* correlates with the pronounced morphological collapse observed by AFM (Table 1).

In the case of PMB, an antibiotic whose main zone of action is the bacterial phospholipid membrane, its structure is extremely crucial. As noted by Sun et al.,^36^ this antibiotic demonstrated a characteristic preference to interact with specific lipid species, especially the cardiolipin and POPG. What is more, the research of Fu et al.^37^ confirmed that polymyxin B absorbs at a shallow level onto the membrane surface. The fatty acyl tails and hydrophobic residues of PMB are known to insert into the lipid tail region. The insertion of PMB has been observed to fill up the lipid packing defect, thereby increasing the lipid tail order, eventually stiffening the membrane and restricting lipid diffusion. In view of the mechanisms described above, which are largely physicochemical, bacterial cells cannot respond significantly to the presence of saponins as well as polymyxin B by remodeling their lipid membranes. And even if the effects on bacterial cells of the two substances tested are therefore similar, they do not weaken each other’s action, and sometimes, as in the case of *E. coli*, the two compounds interact to provide an enhanced membrane remodeling effect.

In comparison to previous studies investigating the antimicrobial properties of *S. mukorossi* extract, our findings provide novel insights into its augmented interaction with the PMB antibiotic against diverse bacterial strains. The observed enhancement in antimicrobial activity and membrane permeability underscores the potential of combining natural plant extracts with conventional antibiotics as a strategy to combat multidrug-resistant bacteria. Further elucidation of the mechanistic pathways underlying these augmented effects is warranted to guide the development of effective therapeutic interventions against bacterial pathogens. In the next stage, the efficacy of the combined treatment should be studied in an *in vivo* model.

## 5 Conclusions

These findings have significant implications for the environment and public health. By reducing the required dosage of PMB, the inclusion of *S. mukorossi* extract offers a promising strategy to mitigate the environmental impact of antibiotic residues and decrease the proliferation of antibiotic resistance genes (ARGs) in soil and water ecosystems. This approach aligns with the urgent need to address antimicrobial resistance (AMR) as both a public health crisis and an environmental pollutant. The use of plant-derived compounds, such as saponins, also exemplifies a sustainable alternative for enhancing antibiotic efficacy while minimizing ecological risks associated with traditional treatments.

Through a series of comprehensive assays, it was demonstrated that the combination of *S. mukorossi* extract with polymyxin B (PMB) antibiotic exhibits enhanced antimicrobial activity compared with PMB alone, as evidenced by improved total membrane permeability across all tested bacterial strains. Presented images from different microscopic techniques highlighted the enhanced efficacy of the combined PMB and SM treatment, manifesting in extensive bacterial cell damage, which is more severe than with either agent alone. The data analysis from AFM calculations highlights that the combined treatment of PMB and SM generally results in more pronounced morphological changes, indicating a higher degree of cellular stress and potential membrane damage, compared to treatment with either agent alone. The variations in response among different bacterial strains suggest species-specific mechanisms in adapting to membrane-disrupting treatments. Moreover, the comprehensive analysis of fatty acid methyl ester (FAME) and lipidomic profiles post-treatment provided additional insights into the biochemical alterations induced by the combination therapy. These results underscore the importance of exploring natural compounds as enhancing agents in antimicrobial therapies, particularly amidst rising concerns of antibiotic resistance.

Overall, this study contributes to advancing environmentally conscious antimicrobial strategies, bridging pharmacological innovation with the global objective of reducing environmental contamination by antibiotics and resistant pathogens. The demonstrated efficacy of *S. mukorossi* as a bioenhancer offers a promising pathway for integrating natural compounds into antimicrobial therapies to combat bacterial pathogens in both environmental and clinical settings.

## Supplementary Material



## Notes

Table S1 a- d. P-values to ANOVA statistical analysis to Figure 1- Tukey’s multiple comparisons test for a-SA, b-SE, c-EC, d-PA; Table S2 a-d. P-values to ANOVA statistical analysis to Figure 2 - Tukey’s multiple comparisons test for a-SA, b-SE, c-EC, d-PA; Figure S1. Lipidomic analysis of bacterial membranes after treatment in comparison to control samples (nontreated bacteria); Gram-positive strains: SA-*Staphylococcus aureus*, SE - *Staphylococcus epidermidis*, and Gram-negative strains: PA-*Pseudomonas aeruginosa*, EC- *Escherichia coli*; treatments: PMB-polymyxin B (100 mg/mL), SM- Sapindus mukorossi (5 mg/mL); treatments provided individually and in conjunction. Lipids abbreviations: PE: Phosphatidylethanolamine; PG: Phosphatidylglycerol; PC: Phosphatidylcholine; CL: Cardiolipin; GL2DAG: Diacylglycerol; LPG: Lysophosphatidyl-glycerol (PDF)

## Abbreviations

SM: *S. mukorossi* extract
bioenhancer PMB: polymyxin B
AFM: atomic force microscopy
TEM: transmission electron microscopy
FAME: fatty acid methyl ester
AMR: antimicrobial resistance
ARB: antibiotic-resistant bacteria
ARGs: antibiotic resistance genes (ARGs)
MTT: 3-(4,5-dimethylthiazol-2-yl)-2,5-diphenyltetrazolium bromide
SA: *Staphylococcus aureus*; SE – *Staphylococcus epidermidis*
PA: *Pseudomonas aeruginosa*
EC: *Escherichia coli*
12:0: Lauric acid (Dodecanoic acid)
14:0: Myristic acid (Tetradecanoic acid)
anteisoC15:0: Anteiso-Pentadecanoic acid (14-Methylpentadecanoic acid)
16:0: Palmitic acid (Hexadecanoic acid)
anteisoC17:0: Anteiso-Heptadecanoic acid (16-Methylheptadecanoic acid)
18:0: Stearic acid (Octadecanoic acid)
18:1: Oleic acid (Octadecenoic acid)
i19:0: Iso-Nonadecanoic acid (17-Methyloctadecanoic acid)
19:0: Nonadecanoic acid (Nonadecylic acid)
20: Arachidic acid (Eicosanoic acid)
PE: Phosphatidylethanolamine
PG: Phosphatidylglycerol PC- Phosphatidylcholine
CL: Cardiolipin GL2DAG- Diglucosyldigliceride
LPG: Lysophosphatidyl-glycerol
