# Supplementary material for: Sapindus mukorossi as a Bioenhancer for the Biocidal Action of Polymyxin B
Source: ACS Omega. 2025 Aug 25;10(35):39658–68. doi: 10.1021/acsomega.5c03035 (PMC12423858; doi:10.1021/acsomega.5c03035)
Supplement: Supplementary file 1 [file ao5c03035_si_001.pdf]

Supplementary Information

Sapindus mukorossi as a bioenhancer for the biocidal action of polymyxin B

Aleksandra Makiej[1], Wojciech Smułek[1], Kamila Dubrowska[2], Adrian Augustyniak[2,3], Przemysław Bernat[4], and Ewa Kaczorek[1]*

[1] Institute of Chemical Technology and Engineering, Faculty of Chemical Technology, Poznan University of Technology, Berdychowo 4, PL-60965 Poznan, Poland

[2] Department of Chemical and Process Engineering, Faculty of Chemical Technology and Engineering, West Pomeranian University of Technology, Szczecin, Piastów Avenue 42, 71-065 Szczecin, Poland

[3] Center for Advanced Materials and Manufacturing Process Engineering (CAMMPE), Piastów Avenue 42, Szczecin, 71-065, Poland

[4] Department of Industrial Microbiology and Biotechnology, Faculty of Biology and Environmental Protection, University of Lodz, 90-136 Lodz, Poland

Supporting Information includes the following content: **Table S1a-d.** P-values to ANOVA statistical analysis to Figure 1- Tukey's multiple comparisons test for a-SA, b-SE, c-EC, d-PA; **Table S2 a-d.** P-values to ANOVA statistical analysis to Figure 2 - Tukey's multiple comparisons test for a-SA, b-SE, c-EC, d-PA; **Figure S1.** Lipidomic analysis of bacterial membranes after treatment in comparison to control samples (non-treated bacteria); Gram-positive strains: SA- *Staphylococcus aureus*, SE- *Staphylococcus epidermidis*, and Gram-negative strains: PA- *Pseudomonas aeruginosa*, EC- *Escherichia coli*; treatments: PMB-polymyxin B (100mg/mL), SM- *Sapindus mukorossi* (5mg/mL); treatments provided individually and in conjunction. Lipids abbreviations: PE: Phosphatidylethanolamine; PG: Phosphatidylglycerol; PC: Phosphatidylcholine; CL: Cardiolipin; GL2DAG: Diacylglycerol; LPG: Lysophosphatidyl-glycerol.

Table S1. P-values to ANOVA statistical analysis to Figure 1

Metabolic activity of selected microbial strains under stress conditions (100% represents the control, non-treated samples); Gram-positive strains: SA- *Staphylococcus aureus*, SE- *Staphylococcus epidermidis*, and Gram-negative strains: PA-*Pseudomonas aeruginosa*, EC- *Escherichia coli*; PMB-polymyxin B (100mg/mL), SM- *Sapindus mukorossi* (5mg/mL).

Table S1a. P-values to ANOVA statistical analysis to Figure 2 - Tukey's multiple comparisons test for SA

Tukey's multiple comparisons test for SA	*Summary*	*Adjusted P Value*
PMB vs. SM	*ns*	*0,7361*
PMB vs. PMB+SM	*ns*	*0,9249*
PMB vs. SA	*	*0,0270*
SM vs. PMB+SM	*ns*	*0,9736*
SM vs. SA	*ns*	*0,1152*
PMB+SM vs. SA	*ns*	*0,0638*

Table S1b. P-values to ANOVA statistical analysis to Figure 2 - Tukey's multiple comparisons test for SE

Tukey's multiple comparisons test for SE	Summary	Adjusted P Value
**PMB vs. SM**	ns	0,4855
**PMB vs. PMB+SM**	ns	0,8806
**PMB vs. SE**	ns	0,0863
**SM vs. PMB+SM**	ns	0,8722
**SM vs. SE**	ns	0,5732
**PMB+SM vs. SE**	ns	0,2375

Table S1c. P-values to ANOVA statistical analysis to Figure 2 - Tukey's multiple comparisons test for PA

Tukey's multiple comparisons test for PA	Summary	Adjusted P Value

**PMB vs. SM**	ns	0,3863
**PMB vs. PMB+SM**	ns	0,6318
**PMB vs. PA**	**	0,0023
**SM vs. PMB+SM**	ns	0,9633
**SM vs. PA**	*	0,0193
**PMB+SM vs. PA**	*	0,0103

Table S1d. P-values to ANOVA statistical analysis to Figure 2 - Tukey's multiple comparisons test for EC

**Tukey's multiple comparisons test for EC**	**Summary**	**Adjusted P Value**
**PMB vs. SM**	ns	0,5457

**PMB vs. PMB+SM**	ns	0,8530
**PMB vs. EC**	ns	0,0571
**SM vs. PMB+SM**	ns	0,9362
**SM vs. EC**	ns	0,3691
**PMB+SM vs. EC**	ns	0,1751

Table S2. P-values to the ANOVA statistical analysis to Figure 2

Total membrane permeability of selected microbial strains under stress conditions in comparison to bacterial non-treated control samples; Gram-positive strains: SA- *Staphylococcus aureus*, SE- *Staphylococcus epidermidis*, and Gram-negative strains: PA- *Pseudomonas aeruginosa*, EC- *Escherichia coli*; PMB-polymyxin B (100mg/mL), SM- *Sapindus mukorossi* (5mg/mL).

Table S2a. P-values to ANOVA statistical analysis to Figure 1 - Tukey's multiple comparisons test for SA

Tukey's multiple comparisons test for SA	Summary	Adjusted P Value
**PMB vs. SM**	**	0,0016
**PMB vs. PMB+SM**	***	0,0005
**PMB vs. SA**	**	0,0067
**SM vs. PMB+SM**	ns	0,6915
**SM vs. SA**	ns	0,6353
**PMB+SM vs. SA**	ns	0,1701

Table S2b. P-values to ANOVA statistical analysis to Figure 1 - Tukey's multiple comparisons test for SE

Tukey's multiple comparisons test for SE	Summary	Adjusted P Value

**PMB vs. SM**	**	0,0030
**PMB vs. PMB+SM**	**	0,0095
**PMB vs. SE**	*	0,0390
**SM vs. PMB+SM**	ns	0,7900
**SM vs. SE**	ns	0,2710
**PMB+SM vs. SE**	ns	0,7273

Table S2c. P-values to ANOVA statistical analysis to Figure 1 - Tukey's multiple comparisons test for PA

**Tukey's multiple comparisons test for PA**	**Summary**	**Adjusted P Value**
**PMB vs. SM**	*	0,0110

**PMB vs. PMB+SM**	*	0,0157
**PMB vs. PA**	ns	0,1306
**SM vs. PMB+SM**	ns	0,9923
**SM vs. PA**	ns	0,3415
**PMB+SM vs. PA**	ns	0,4686

Table S2d. P-values to ANOVA statistical analysis to Figure 1 - Tukey's multiple comparisons test for EC

Tukey's multiple comparisons test for EC	Summary	Adjusted P Value
**PMB vs. SM**	*	0,0379
**PMB vs. PMB+SM**	ns	0,3888

**PMB vs. EC**	ns	0,8852
**SM vs. PMB+SM**	ns	0,3772
**SM vs. EC**	*	0,0141
**PMB+SM vs. EC**	ns	0,1522

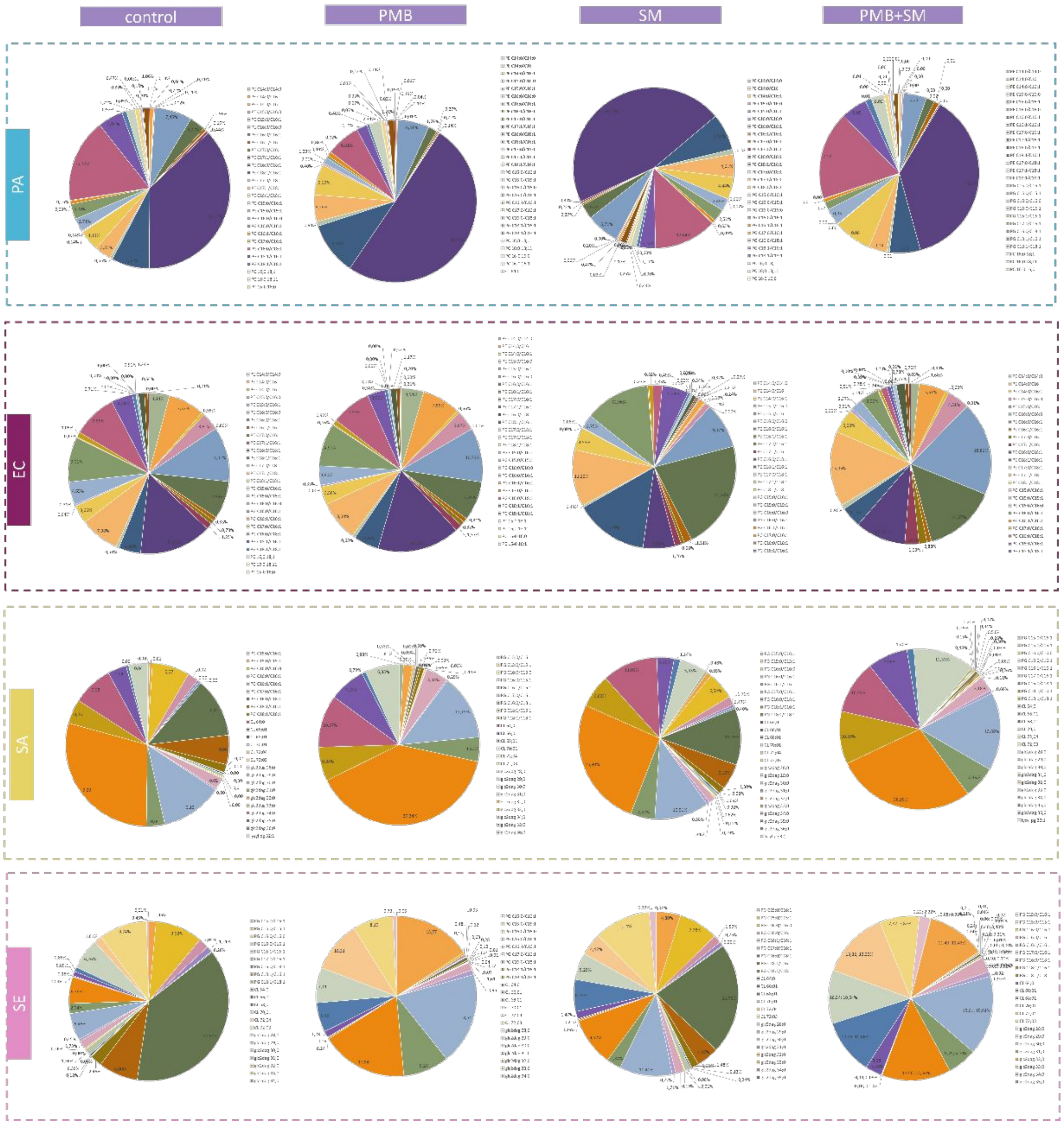

Figure S1. Lipidomic analysis of bacterial membranes after treatment in comparison to control samples (non-treated bacteria); Gram-positive strains: SA-*Staphylococcus aureus*, SE-*Staphylococcus epidermidis*, and Gram-negative strains: PA-*Pseudomonas aeruginosa*, EC-*Escherichia coli*; treatments: PMB-polymyxin B (100mg/mL), SM- *Sapindus mukorossi* (5mg/mL); treatments provided individually and in conjunction. Lipids abbreviations: PE:

Phosphatidylethanolamine; PG: Phosphatidylglycerol; PC: Phosphatidylcholine; CL: Cardiolipin; GL2DAG: Diacylglycerol; LPG: Lysophosphatidyl-glycerol.
